# Comparative study of CAD/CAM reconstruction and miniplates for patient-specific fixation in LCL-type mandibular reconstruction

**DOI:** 10.3389/fonc.2024.1438269

**Published:** 2024-09-11

**Authors:** Philipp Lampert, Jakob Fenske, Jonas Wüster, Steffen Koerdt, Kilian Kreutzer, Philipp Ruf, Sara Checa, Max Heiland, Claudius Steffen, Carsten Rendenbach

**Affiliations:** ^1^ Charité - Universitätsmedizin Berlin, Corporate Member of Freie Universität Berlin and Humboldt-Universität zu Berlin, Department of Oral and Maxillofacial Surgery, Berlin, Germany; ^2^ Berlin Institute of Health at Charité - Universitätsmedizin Berlin, Julius Wolff Institute, Berlin, Germany

**Keywords:** mandibular reconstruction, patient-specific, 3D-printing, titanium plate, miniplate, fibula flap, pseudarthrosis, CAD/CAM

## Abstract

**Objective:**

Miniplates offer superior clinical handling and facilitate postoperative removal after mandibular reconstruction but unfavorable load distribution under high stress has been shown. This study aimed to compare the clinical outcome of patient-specific 3D-printed (PS-3D) titanium miniplate with reconstruction plate fixation in three-segmental LCL-type reconstructions for the first time.

**Methods:**

Patients undergoing three-segmental LCL-type mandibular reconstruction after malignant tumor resection between April 2017 and July 2023 were analyzed in a retrospective single-center study. Inclusion criteria were primary reconstruction using a fibula free flap and PS-3D titanium mini- or reconstruction plate fixation. Complication rates were recorded and analyzed within 6 months after surgery using the N – 1 Chi^2^- and unequal variance t-test.

**Results:**

38 patients (10 females, 28 males; mean age 61.4 ± 7.6 years) met the inclusion criteria. In 14 patients (36.8%) miniplates were used in the anterior region. Rates of fixation failure, plate exposure, incomplete osseous union, wound infection, soft tissue, and overall complications did not differ significantly between the two plate systems.

**Conclusion:**

Complication rates did not differ significantly between PS-3D mini- and reconstruction plates in three-segmental LCL-type mandibular reconstructions. Given their advantages in clinical handling and postoperative removal, PS-3D miniplates can be a viable alternative also in larger mandibular reconstructions.

## 1 Introduction

Mandibular segmental resection is the first-line treatment for oral carcinomas infiltrating the mandible (1). To restore chewing function, osseous free flap reconstruction followed by dental implant placement remains the gold standard (2). While superior to other reconstructive methods, post-operative complications remain, most notably osseous non-union, plate exposure and soft tissue complications (3, 4).

A significant risk factor for these complications are large mandibular defects, often requiring multi-segment flaps for reconstruction (5, 6). These extensive reconstructions necessitate a plate system capable of withstanding the increased mechanical stress of fixating multiple segments over a longer distance, while also promoting bone regeneration through beneficial strain (7, 8).

Recently, the use of patient-specific 3D-printed (PS-3D) plates has become increasingly common in mandibular reconstruction due to more predictable plate design and simplified surgical handling (9, 10). Nevertheless, plate-related complication rates have not improved significantly (4). In fact, the increased stiffness of PS-3D reconstruction plates is assumed to cause higher rates of osseous non-union (4, 11).

PS-3D miniplates were introduced by our group in 2022, to address some of the potentially complication-inducing properties of PS-3D reconstruction plates by allowing higher inter-osteotomy movements (IOM) and facilitating removability after surgery (8, 12, 13). Due to the reduced size of miniplates and the use of monocortical screws, plate removal can be performed via an intraoral approach in an outpatient setting with dental implantation in the same surgery resulting in significantly reduced treatment time and cost (12). In single-segment reconstructions, Ruf et al. demonstrated increased beneficial mechanical straining when using PS-3D miniplate fixation in the canine region over PS-3D reconstruction plate fixation (14). However, miniplates exhibited uneven load distribution when used for fixation at the mandibular angle, an area known to be associated with higher stress than the symphysis (15). To overcome this problem, a combined plate system for single-segment mandibular reconstructions using fibula free flaps was proposed: A shortened reconstruction plate at the mandibular angle and a pair of miniplates at the symphysis (16).

To date, this combined plate system has clinically only been evaluated for single- and two-segmental reconstructions where a tendency towards reduced complication rates compared to single PS-3D reconstruction plate fixation was registered (17). However, its behavior in high-stress LCL-type (18) reconstructions has not yet been evaluated clinically or biomechanically and is therefore of great interest.

We hypothesized that a combined PS-3D mini- and reconstruction plate osteosynthesis would not lead to increased complication rates compared to single reconstruction plate fixation in three-segmental LCL-type mandibular reconstructions using fibula free flaps.

## 2 Methods

### 2.1 Study design

This retrospective single-center cohort study was designed at the department of Oral and Maxillofacial Surgery at Charité – Universitätsmedizin Berlin. Patients operated between April 2017 and July 2023 were deemed eligible for study enrollment. Follow-up documentation was analyzed until January 2024. Ethical approval was obtained from the local ethics committee (EA2/138/18).

### 2.2 Inclusion and exclusion criteria

Inclusion criteria were fixation using a single PS-3D reconstruction plate bridging the entire three-segmental LCL-type defect or two shortened reconstruction plates at the mandibular angle in combination with PS-3D miniplates at the anterior region. Reconstructions involving the mandibular ramus were not included as biomechanical behavior and stress exhibited on the plates was assumed to differ significantly. The minimum follow up period was 6 months after surgery. To establish a homogenous cohort, only patients who received a fibula free flap as primary reconstruction following malignant tumor resection were included, as other common indication such as osteoradionecrosis are associated with poor clinical outcome themselves. We reviewed but excluded patients who suffered a flap loss within the follow-up period.

### 2.3 Procedures

The computer aided design/computer aided manufacturing (CAD/CAM) workflow of PS-3D plate design has previously been described in detail by our department (12, 17). All plates were manufactured by KLS Martin SE & Co. KG (Tuttlingen, Germany) using a titanium 3D-printing process. Reconstruction plates were designed with a thickness of 2.0 mm and height of 8.0 mm, mini plates with a thickness of 1.0 mm and height of 5.0 mm. Plate length was adjusted individually for each case. Miniplates were fixated with four 2.0 x 7.0 mm monocortical screws per plate, used in pairs at the corpus-symphysis intersegmental gaps and supported by two 6-hole reconstruction plates at the distal intersegmental gaps (Group 1) (**Table 1**). The reconstruction plates were fixated to the mandibular stumps using 2.0 mm bicortical screws while 7.0 mm monocortical screws were used for fixation of the free flap to protect the vascular pedicle (Group 2) (**Table 1**). For the first postoperative week, low molecular weight heparin (Fraxiparine 0.3 ml twice a day) was administered. Patients that received radiotherapy were treated according to the current German Cancer Guideline for Oral Cavity Cancer (1). The total dose administered was between approximately 70 Gy.

**Table 1:** 3D planning, clinical execution, and description of the two plate systems compared in this study.

Group 1: Reconstruction + miniplates	Group 2: Single reconstruction plate
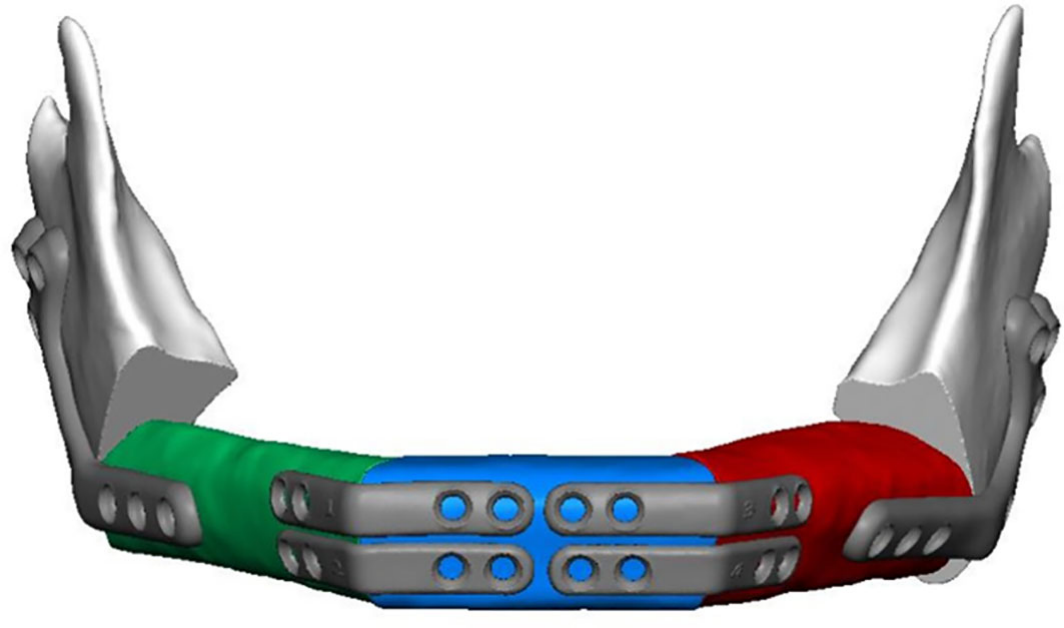	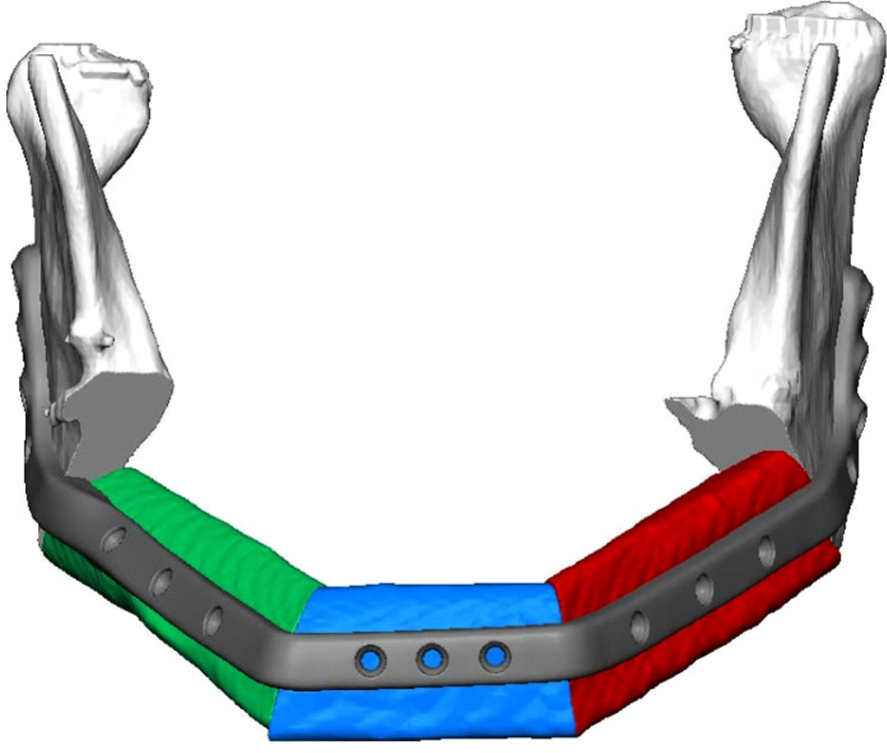
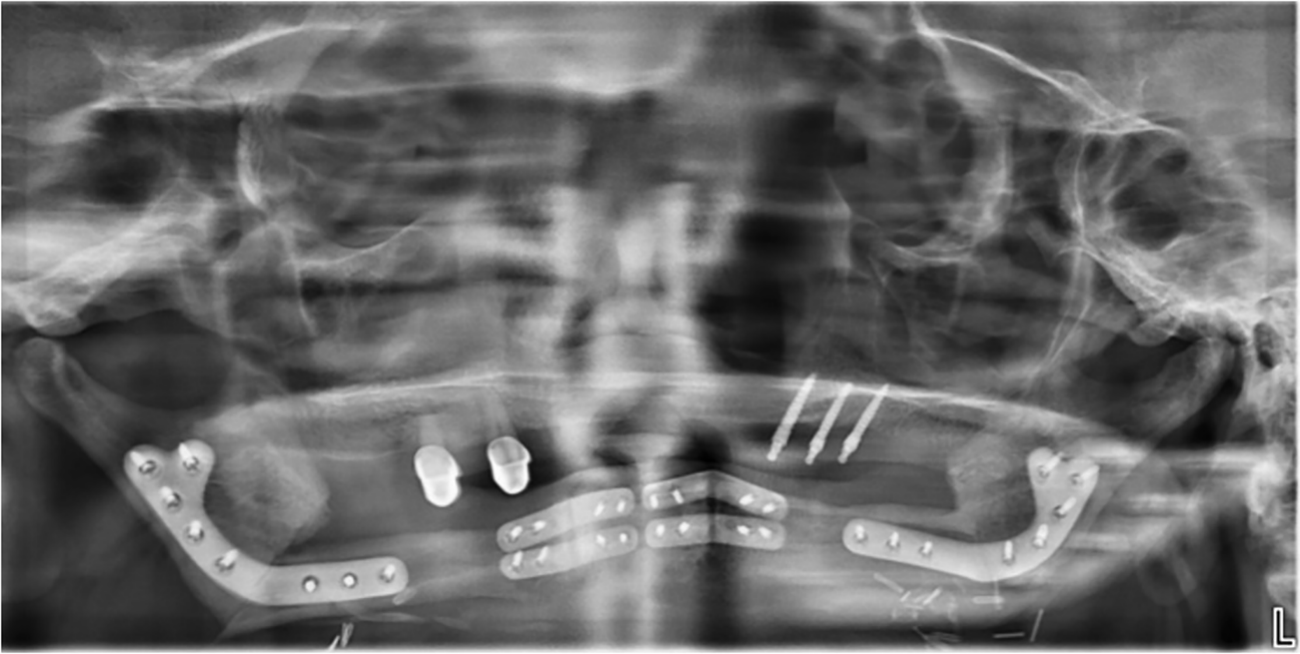	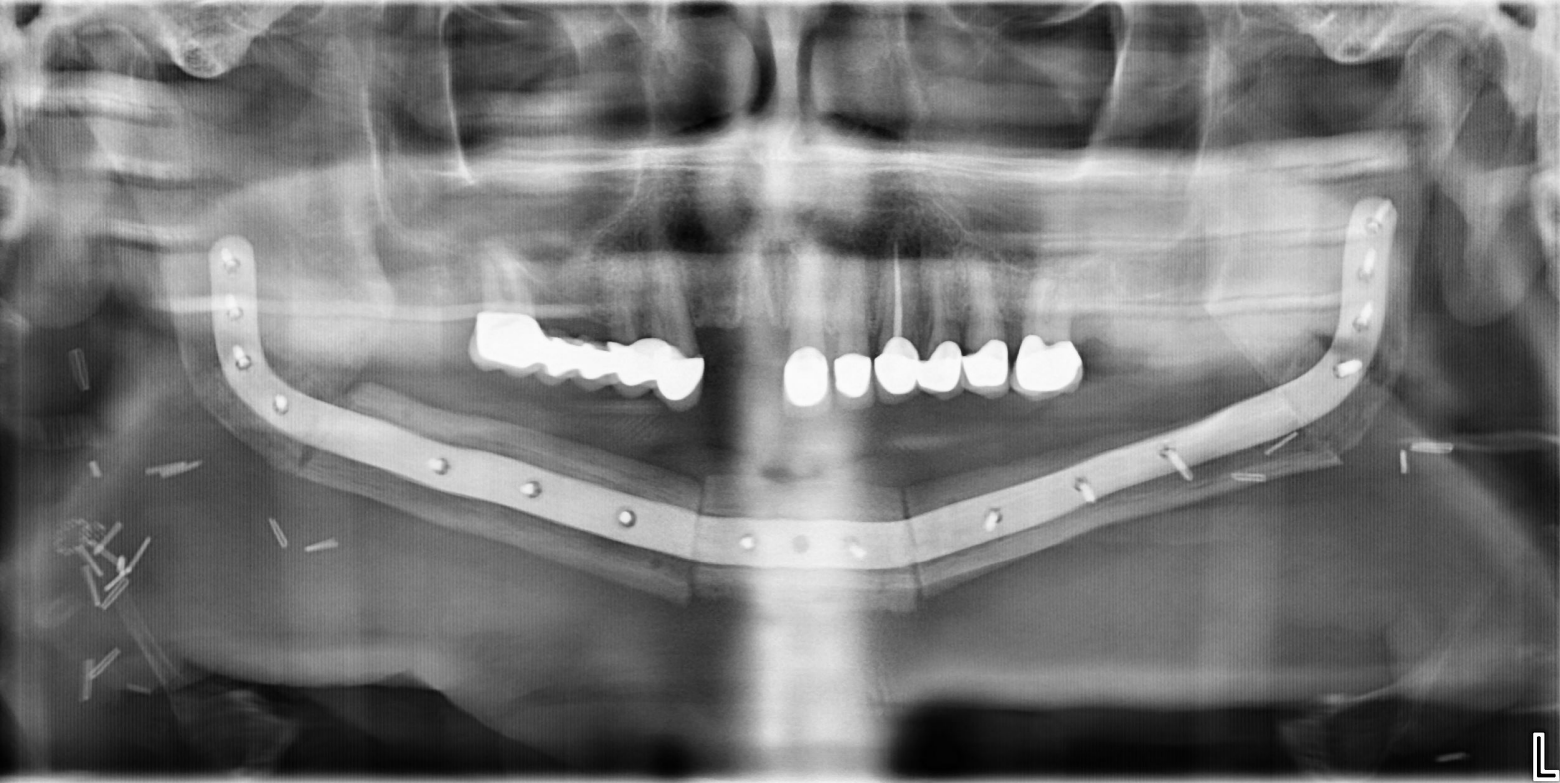
Combination of short PS-3D reconstruction plates at the distal intersegmental gaps and PS-3D miniplates at the anterior intersegmental gaps	Single PS-3D reconstruction plate spanning all intersegmental gaps

PS-3D, patient-specific 3D-printed.

### 2.4 Data acquisition

All data was captured and managed using REDCap electronic data capture tools hosted at Charité – Universitätsmedizin Berlin (RRID: SCR_003445) (19, 20). Medical charts of all patients were screened for patient- and disease-related information: age at surgery, sex, body mass index (BMI), nicotine consumption, alcohol abuse, atherosclerosis, adjuvant radio- and chemotherapy. Information about the fixation system and use of an intraoral skin paddle was taken from the surgeon’s report. The follow-up documentation was screened for any of the predefined outcomes within a follow-up period of 6 months (**Table 2**). Radiographic images were taken 6 months after surgery and analyzed for osseous union.

**Table 2:** Pre-defined diagnostic criteria for each outcome.

Outcome	Diagnostic criteria
Any complication	- any of the complications listed below
Fixation failure	- plate loosening - plate fracture
Plate exposure	- intraoral plate exposure - extraoral plate exposure
Incomplete osseous union	- ≥ 1 intersegmental gap with less than 50% radiographic ossification at least 6 months after surgery (diagnosed in CBCT, CT or OPT scans with decreasing priority)
Wound infection	- pus - infectious fistula - abscess formation
Soft tissue complication	- wound healing disorder - wound dehiscence - partial skin necrosis - plate exposure - bone exposure - wound infection (see criteria above)

CT, computer tomography; CBCT, cone-beam CT; OPT, panoramic radiograph.

One fulfilled criterion sufficed to record the respective outcome.

### 2.5 Statistical analysis

Data engineering and statistical analysis was performed using the Python Programming Language version 3.11.5 (RRID: SCR_008394) and the Scipy Stats module (21–23). There was no missing data among predictor variables. Records with missing data in outcome variables were excluded in the respective analyses. Numeric variables were tested for normality using the Shapiro-Wilk-Test and analyzed for significant differences between the two plate groups using the unequal variance t-test (24). Nominal variables were tested for significance using the N - 1 Chi^2^-test as recommended by Campbell (25). Our study’s level of significance was set at p ≤ 0.05. In addition, inclusion of the null value in the 95%-confidence interval (CI) of an odds ratio (OR) was recorded as non-significant, while non-inclusion was recorded as significant.

## 3 Results

### 3.1 Patient inclusion process

**Figure 1:**
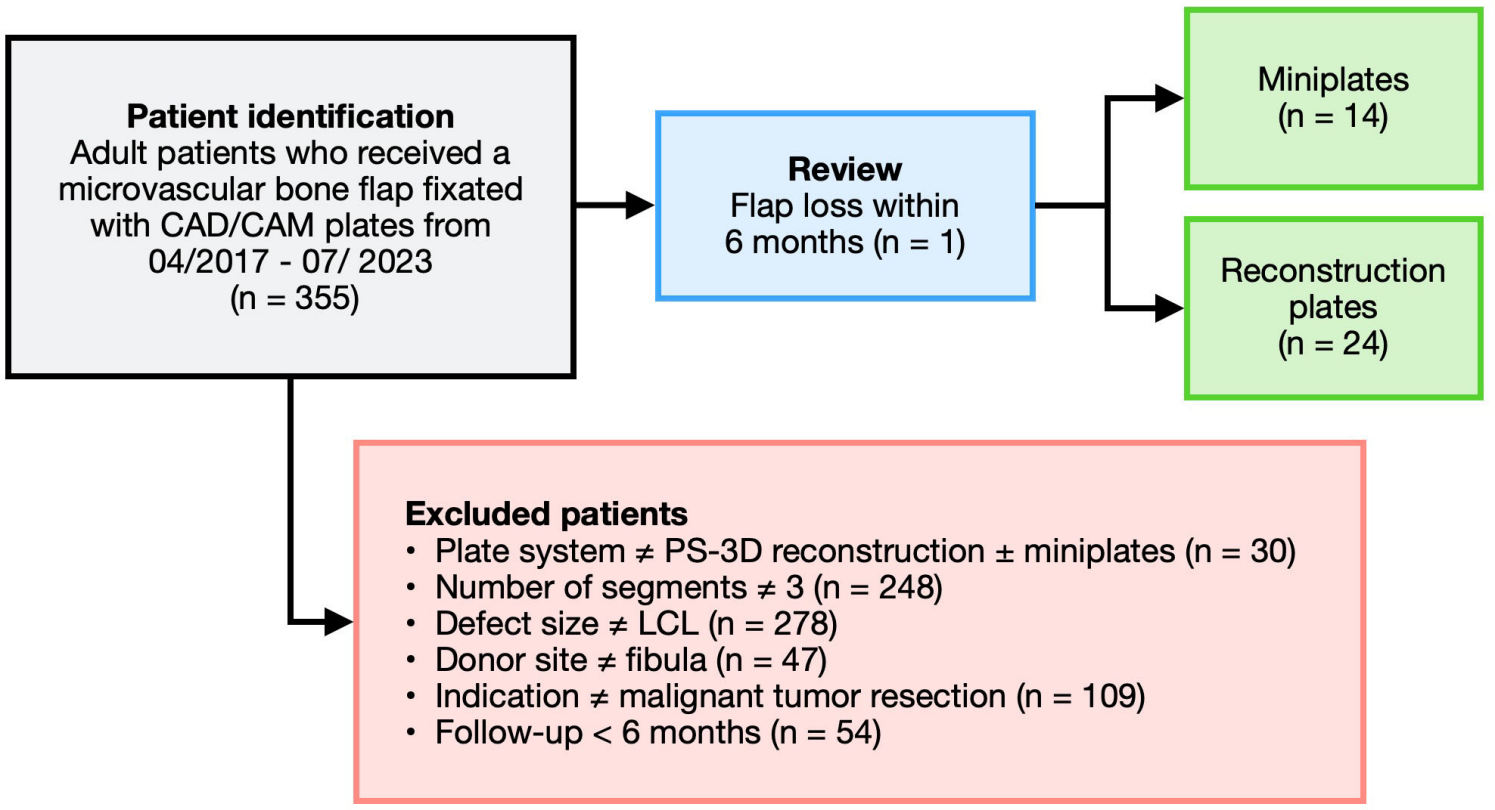
Visualization of the patient inclusion process.

355 patients were initially identified as eligible for study enrollment. Patients were excluded as per the previously described criteria. Of the remaining 39 patients, 1 (2.6%) patient belonging to the reconstruction plate group suffered a flap loss within the follow-up period. This case was excluded, resulting in a study population of 38 patients (**Figure 1**).

### 3.2 Baseline characteristics

The baseline characteristics of the study population are shown in (**Table 3**). All numeric variables were normally distributed. The patient collective was homogenous and differed significantly only in defect length between the two plate groups (p = .038): Patients who received the combined plate system had a mean defect length of 129.6 mm (± 17.3) while the other group had a slightly lower mean defect length of 113.5 mm (± 26.5). An intraoral skin paddle was used slightly less often with the combined plate system (42.9% vs. 66.7%, p = .157) while adjuvant chemotherapy was more common in the reconstruction plate group (50.0% vs. 28.6%, p = .203).

**Table 3:** Baseline characteristics of the study population.

	Combined plates	Reconstruction plate	p-value	Overall
	n = 14 (36.8%)	n = 24 (63.2%)		N = 38 (100%)
**Age (years)**			.594	
Mean ± SD	60.6 ± 7.6	62.0 ± 7.8		61.4 ± 7.6
**Sex**			.606	
Female	3 (21.4)	7 (29.2)		10 (26.3)
Male	11 (78.6)	17 (70.8)		28 (73.7)
**BMI (kg/m^2^)**			.917	
Mean ± SD	23.7 ± 2.3	23.6 ± 4.7		23.6 ± 3.9
**Nicotine abuse**			.246	
Yes	6 (42.9)	15 (62.5)		21 (55.3)
No	8 (57.1)	9 (37.5)		17 (44.7)
**Alcohol abuse**			.581	
Yes	4 (28.6)	9 (37.5)		13 (34.2)
No	10 (71.4)	15 (62.5)		25 (65.8)
**Atherosclerosis**			.441	
Yes	2 (14.3)	6 (25.0)		8 (21.1)
No	12 (85.7)	18 (75.0)		30 (78.9)
**Radiotherapy**			.748	
Yes	8 (57.1)	15 (62.5)		23 (60.5)
No	6 (42.9)	9 (37.5)		15 (39.5)
**Chemotherapy**			.203	
Yes	4 (28.6)	12 (50.0)		16 (42.1)
No	10 (71.4)	12 (50.0)		22 (57.9)
**Intraoral skin paddle**			.157	
Yes	6 (42.9)	16 (66.7)		22 (57.9)
No	8 (57.1)	8 (33.3)		16 (42.1)
**Defect length (mm)**			.038	
Mean ± SD	129.6 ± 17.3	113.5 ± 29.3		119.4 ± 26.5

SD, standard deviation; BMI, Body Mass Index.

### 3.3 Bivariate analysis

Complication rates did not differ significantly between the two plate groups. One case of fixation failure occurred in the combined plates group due to a loosening of miniplates at the anterior mandibular segment. Imaging data from within the follow-up period was missing for 5 patients, resulting in a reduced number of cases analyzed for osseous union. Plate exposure occurred intraorally only (**Table 4**).

**Table 4:** Results of bivariate analyses between the two plate systems and all outcomes.

	Combined plates	Reconstruction plate	p-value	OR [95%-CI]	Overall
	n = 14 (36.8%)	n = 24 (63.2%)			N = 38 (100%)	
**Any complication**			.441	2.00 [0.34; 11.62]	
Yes	12 (85.7)	18 (75.0)			30 (78.9)
No	2 (14.3)	6 (25.0)			8 (21.1)
**Fixation failure**			.190	5.44 [0.21; 143.10]	
Yes	1 (7.1)	0 (0.0)			1 (2.6)
No	13 (92.9)	24 (100.0)			37 (97.4)
**Plate exposure**			.260	2.25 [0.55; 9.17]	
Yes	6 (42.9)	6 (25.0)			12 (31.6)
No	8 (57.1)	18 (75.0)			26 (68.4)
**IOU overall**			.510	1.62 [0.39; 6.68]	
Yes	9 (64.3)	10 (52.6)			19 (57.6)
No	5 (35.7)	9 (47.4)			14 (42.4)
**Wound infection**			.122	3.00 [0.74; 12.13]	
Yes	7 (50.0)	6 (25.0)			13 (34.2)
No	7 (50.0)	18 (75.0)			25 (65.8)
**Soft tissue complication**			.748	0.80 [0.21; 3.06]	
Yes	8 (57.1)	15 (62.5)			19 (57.6)
No	6 (42.9)	9 (37.5)			14 (42.4)

OR, odds ratio; CI, confidence interval; IOU, incomplete osseous union.

## 4 Discussion

This study is the first to analyze the postoperative outcome of PS-3D miniplate fixation in three-segmental LCL-type mandibular reconstructions. We chose a combined system of two six-hole PS-3D reconstruction plates at both distal intersegmental gaps with PS-3D miniplates at the anterior gaps based on biomechanical findings by Ruf et al. (**Figure 1**) (14). This combined plate system had previously shown a tendency towards reduced complication rates in mandibular single- and two-segmental reconstructions and offers superior clinical handling when compared to single PS-3D reconstruction plate fixation (17).

Patient characteristics did not significantly differ between the two plate groups, except for defect length, which was longer in the combined plate group (129.6 ± 17.3 vs. 113.5 ± 29.3 mm, p = .038). While statistically significant, the absolute mean difference of 16.1 mm is relatively small and, if anything, may have slightly disadvantaged the new combined plate system due to the increased defect length.

In our study, the combined plate system was not inferior compared to single reconstruction plate fixation, as complication rates did not differ significantly. While higher fixation failure (7.1% vs. 0.0%, p = .190) and wound infection rates (50.0% vs. 25.0%, p = .122) were observed, this may be related to our study’s limited sample size of 38 patients. It does not imply an inferiority of miniplates as the difference was not significant and may be related to randomness. However, further biomechanical and clinical analyses are recommended.

The present study’s flap loss rate of 2.6% is lower than previously described rates ranging from 4.7% to 9.4%, although our follow-up time of 6 months was shorter than that of some other studies (3, 26). By only including patients who underwent fibula free flap reconstruction following malignant tumor resection, we avoided potential confounders arising from different flap types and surgical indications. This established a homogenous cohort of 38 patients which did not differ significantly between the two plate groups. An intraoral skin paddle was used slightly less often with the combined system (42.9% vs. 66.7%, p = .157). This difference is mainly due to our team’s recent shift in operating technique, favoring the use of muscle tissue over a skin island to avoid a bulky soft tissue mass in the oral cavity. As miniplates were introduced later, a skin paddle was used less often in those surgeries.

### 4.1 Reconstruction versus miniplates

Several studies have previously compared conventional mini- and reconstruction plates, recently summarized in a meta-analysis by Sobti et al. (26). Their findings showed plate exposure and fixation failure rates to be significantly higher among conventional miniplates which contrasts with our findings showing no significant differences between PS-3D mini- and reconstruction plates (p = .260 and p = .190). However, their rates of 32.5% for conventional mini- and 18.8% for conventional reconstruction plates were lower than our plate exposure rates of 42.9% and 25.0% for PS-3D mini- and reconstruction plates, respectively. This is in line with prior studies reporting on moderately increased complication rates for PS-3D plates (4, 27). While there was no case of extraoral plate exposure in the present study, the increased rate of intraoral plate exposure among miniplates can be attributed to a less frequent use of skin paddles by our team. In our experience, however, initial plate exposure of CAD/CAM miniplates typically does not lead to additional complications like osseous non-union or wound infection. Furthermore, this study did not compare conventional with PS-3D plates, and differences in complication definition and recording prevent cross-study comparisons.

Ultimately, while complication rates seem to be significantly higher among conventional miniplates compared to conventional reconstruction plates, the same cannot be said for the combined system of PS-3D mini- and reconstruction plates analyzed in this study, as complication rates did not differ significantly compared to the reconstruction plate group. This is particularly beneficial since miniplates by design offer considerable advantages over reconstruction plates in clinical handling. Due to their reduced size, plate removal can be performed via an intraoral approach in an outpatient setting with dental implantation in the same surgery (28). This contrasts with reconstruction plate fixation, where achieving a similar outcome would often require intraoperative plate cutting and hospitalization, resulting in considerably higher costs (17).

### 4.2 Surgical handling of miniplates

The fixation failure rate of 2.6% remains lower than previously reported rates between 7.7% and 12.4% (3, 29). It resulted from one case of plate loosening from the combined plate group, were both miniplate fixations at the anterior mandibular region loosened and were slightly dislocated. Our experience has shown that correct handling of miniplate fixation is highly relevant and can prevent post-operative plate loosening. Cutting guides need to be positioned with great precision and continuous additional rinsing during drilling is essential to prevent heat damage in the drilling holes. We further recommend pre-fixation of all plates at the harvesting side prior to vessel ligation without fully tightening any screw. Only once the 3-segmental fibula free flap can be placed in the desired position between the residual mandibular stumps without putting stress on plates or screws should the three posterior screws be inserted in the mandibular stump on each side and all other screws fixated definitively. Compression with 2 hands from the lateral side during anterior screw fixation is sometimes necessary to avoid rotation and consequently mispositioning of the segments. Only a very thin muscle cuff and periosteum should remain on top of the lateral fibula during flap harvesting to enable sufficient miniplate fixation with mono-cortical screws in a load-sharing manner through frictional loading between the fibula segments and fixation plates.

### 4.3 Bone healing

Bone regeneration is essential to prevent osseous non-union but has been shown to be sensitive to suboptimal mechanical conditions (30, 31). Unfavorable inter-osteotomy movements (IOMs) due to inadequate load distribution may therefore lead to an incomplete osseous union, in turn resulting in chronic overload on the osteosynthesis material. This can potentially lead to fixation failure, especially if patients regain their full bite force. Despite theoretical advantages of PS-3D miniplates in bone regeneration in single-segment reconstructions (14), the present clinical study revealed no difference in osseous non-union rates compared to PS-3D reconstruction plate fixations (64.3% vs. 52.6%, p = .510) in LCL-type reconstructions. While our observed rates are in line with studies by Knitschke et al. for conventional and PS-3D reconstruction plates (27, 29), Kreutzer et al. found a significantly reduced osseous non-union rate for the combined plate system in single- and two-segmental reconstructions (6.7% vs. 46.2%, p = .029) (17). Given that LCL-type reconstructions usually result in postoperative toothlessness and thus permanently reduced bite forces, miniplates would have been assumed to be particularly beneficial in such low-stress scenarios as this has been shown to result in increased beneficial straining in single-segment reconstructions (14). High rates of incomplete osseous union for miniplate fixations, as seen in the present study, are therefore unexpected but may be related to the increased instability of three-segmental LCL reconstructions. Whether a load-bearing reconstruction plate is beneficial due to its rigid fixation remains to be investigated by future biomechanical studies, as such an effect could not be proven in the present clinical study.

Nevertheless, it should not be overlooked that patients requiring LCL-type reconstructions due to malignant tumors are more likely to also receive adjuvant radiotherapy, a known risk factor inhibiting osseous union (27). Furthermore, we screened patients for osseous union 6 months after surgery, limiting comparability to other studies with a longer follow-up period, as Knitschke et al. have shown osseous union rates to improve considerably over time (27). Further biomechanical studies employing bite force analyses are needed to analyze how mechanical strains in LCL-type reconstructions can be improved. Existing biomechanical analyses, focused only on single-segment reconstructions, may not accurately reflect the mechanical stresses present in more extensive defects (32).

### 4.4 Study limitations

Our study is associated with some limitations that should be mentioned. While our focus on a homogenous cohort strengthens the internal validity of our findings, it comes at the cost of excluding other relevant surgical indications such as osteoradionecrosis and flaps other than fibula free flaps. Additionally, three-segmental LC-type reconstructions and those including the mandibular ramus were excluded from the present study, as these reconstructions are assumed to differ substantially from the studied LCL-type reconstructions regarding the mechanical stress exhibited on plate system and transplant. We chose a follow-up time of 6 months as most complications tend to occur within this time frame and relatively few patients had to be excluded due to an incomplete follow-up. However, long-term complications occurring later than 6 months post-surgery were not captured and should be evaluated in future studies. Ultimately, our study’s retrospective design comes with inherent limitations, only allowing limited generalization of the results.

## 5 Conclusion

Our study found no significant differences in complication rates between PS-3D mini- and reconstruction plates. Given the established benefit of easier postsurgical miniplate removal in the anterior mandibular region, clinical superiority over single PS-3D reconstruction plate fixation can be inferred. Further research is needed to understand load distribution and failure mechanisms specifically in multi-segment mandibular reconstructions, as biomechanical conditions may differ considerably from single-segment reconstructions. Perioperative functional analyses can thereby help identify strategies to improve osseous healing in large mandibular reconstructions.

## Data Availability

The raw data supporting the conclusions of this article will be made available by the authors, without undue reservation.
